# The Prince at Bristol and Cardiff

**Published:** 1921-06-18

**Authors:** 


### THE PRINCE AT BRISTOL AND CARDIFF.

The tour of the Prince of Wales in Bristol and South Wales included some interesting hospital proceedings, though in the former city our correspondent writes that the Prince was given such a terrific programme that he barely had time to lay the foundation-stone of the new building of the Homœopathic Hospital, which is being erected as a memorial to Captain Bruce Melville Wills by his father. The building designed by Messrs. Oatley and Lawrence will occupy an ideal site on the summit of Cotham Hill. Despite the fact that the length of the Bristol programme had made him late, the Prince did not omit when at Cotham House to review and talk to the disabled ex-Service men who were present.

If the chief ceremonial event at Cardiff was the Prince's installation as Chancellor of the University of Wales, hardly less important was the afternoon ceremony on the same day, when the Prince laid the foundation-stone of the Department of Public Health and the School of Preventive Medicine. Then the Prince also opened the new physiological buildings of University College, which were begun in 1915. It was a great occasion for the Welsh Medical School, a foundation which has been established, in spite of many difficulties, through the devoted work and generosity of such men as the late Colonel Bruce-Vaughan, Sir William James Thomas, and Lord Glanely. Sir W. J. Thomas indeed is virtually the donor of the National School of Medicine, and it was he who handed to the Prince the deed of gift for presentation to the President of the College. In that symbolic act a long story is represented, for, though University College was founded only in 1883, the foundation of a Medical School has meant a long struggle. In 1906 the University of Wales by a supplemental charter became empowered to confer degrees in medicine and surgery, but even then the course of study had to be partly taken elsewhere. In 1909 a small Treasury grant was obtained; in 1910 a chair of pathology was founded, and side by side with these events King Edward VII. Hospital, Cardiff, was growing so as to take its due place in the work of the Medical School. It fell to the late Colonel Bruce-Vaughan to announce the first of Sir W. J. Thomas's munificent gifts. The Department of Physiology and the School of Preventive Medicine are the monuments of his generosity. By the autumn the Welsh National Medical School, which the Prince has now inaugurated, will form a self-contained part of the College, able to supply all the needs of its students, who now number 280.

